# How participatory is parental consent in low literacy rural settings in low income countries? Lessons learned from a community based study of infants in South India

**DOI:** 10.1186/1472-6939-12-3

**Published:** 2011-02-15

**Authors:** Divya Rajaraman, Nelson Jesuraj, Lawrence Geiter, Sean Bennett, Harleen MS Grewal, Mario Vaz

**Affiliations:** 1 Division of Epidemiology, St John's Research Institute, Bangalore, India; 2 Formerly with AERAS Global TB Vaccine Foundation, Maryland, USA; 3 The Gade Institute, Section for Microbiology and Immunology, University of Bergen, Bergen, Norway; 4 Department of Microbiology and Immunology, Haukeland University Hospital, Bergen, Norway

## Abstract

**Background:**

A requisite for ethical human subjects research is that participation should be informed and voluntary. Participation during the informed consent process by way of asking questions is an indicator of the extent to which consent is informed.

**Aims:**

The aims of this study were to assess the extent to which parents providing consent for children's participation in an observational tuberculosis (TB) research study in India actively participated during the informed consent discussion, and to identify correlates of that participation.

**Methods:**

In an observational cohort study of tuberculosis in infants in South India, field supervisors who were responsible for obtaining informed consent noted down questions asked during the informed consent discussions for 4,382 infants who were enrolled in the study. These questions were post-coded by topic. Bivariate and multivariate analysis was conducted to examine factors associated with asking at least one question during the informed consent process.

**Results:**

In total, 590 out of 4,382 (13.4%) parents/guardians asked any question during the informed consent process. We found that the likelihood of parents asking questions during the informed consent process was significantly associated with education level of either parent both parents being present, and location.

**Conclusions:**

The findings have implications for planning the informed consent process in a largely rural setting with low levels of literacy. Greater effort needs to be directed towards developing simple participatory communication materials for the informed consent process. Furthermore, including both parents in a discussion about a child's participation in a research study may increase the extent to which consent is truly informed. Finally, continuing efforts need to be made to improve the communication skills of research workers with regard to explaining research processes and putting potential research participants at ease.

## Background

The aims of this study were to assess the extent to which parents providing consent for children's participation in an observational tuberculosis (TB) research study in India actively participated during the informed consent discussion, and to identify correlates of that participation.

An essential requirement for ethical research is that participation in trials should be informed and voluntary. However, operationalization of the principle of informed consent in community settings with low levels of education and research literacy can pose challenges, especially since standardized consent protocols may not be applicable across different social, cultural and economic settings [1-3]. Medical research in children poses particular concerns for informed consent because of children's status as a vulnerable population, legally incompetent to make decisions about their own participation in research studies [4]. A child's parent or legal guardian usually serves as a proxy consenter, making decisions about research participation on behalf of the child. In many countries, including India, only one parent/guardian's signature is legally required to enrol a child in a study. However, household decision-making processes and gender differences in attitudes towards health research mean that researcher decisions about which parent to approach for consent can have implications for the process of informed consent in terms of the amount of information requested by potential research subjects, as well as ultimate decisions about whether or not to participate [5-8].

Researchers have started to work towards developing cross-cultural understandings of research ethics by examining research literacy and the informed consent process in sub-Saharan Africa, North Africa and Asia [5-7,9-14]. A range of issues have been identified with respect to the transferability of a code of medical ethics across different settings. These include questions around the relative importance of community versus individual consent [6,15-17], and whether consent can be truly informed amongst populations with low research literacy; for example, it may not be easy to impart key concepts of clinical research such as randomization, placebo and future societal good versus current individual benefits [18]. A further concern is that organizations that conduct research may also provide health care services which create a high level of trust; this can be a barrier to healthy skepticism necessary for asking questions about the aims and consequences of a research project [9,19]. A related issue is the common phenomenon of 'therapeutic misconception', where research subjects incorrectly interpret the primary purpose of a clinical trial to be therapeutic rather than experimental, sometimes resulting in false hopes about individual outcomes [20,21].

Recent reviews of the research process in developing country settings have endorsed the importance of community-level participation and subsequent individual consent for research, particularly in rural areas where community level decision-making is more prevalent compared to urban areas [3,5,6,21]. In line with these recommendations, most community research studies in developing countries are preceded by community-wide information sessions and handouts as a first step for raising awareness of the research study and procedures. However, understanding of the nature and purpose of a research study is often low, even where informed consent has been obtained through standardized and approved procedures [22]. Moreover, few questions may be asked during the informed consent process although participants continue to have questions about the study and are provided ample opportunities to clarify any doubts or seek further information [18].

In order to ensure ethical research across different social and cultural contexts, there is a need for more empirical evidence to understand the informed consent process. This is particularly important in developing countries, where the enforcement of codes of medical research ethics may be weaker. The analysis reported in this paper contributes to debates about how to increase researchers' engagement with research participants, by focusing on the process of consent for enrolling children in a community-based prospective study to assess the incidence of pulmonary tuberculosis (TB) in infants in rural South India. We were interested in examining the extent to which individuals giving consent actively participated in the consent process, and identifying the correlates of participation. The proxy measure for participation was whether any question was asked during the consent discussion.

## Methods

### Study Setting

The study reported here analyses data collected during the informed consent discussions for an observational, cohort study that was designed to assess the incidence of TB in infants enrolled within 2 weeks of birth and followed up for a period of 2 years. The study was conducted in the Palamaner area of Chittoor district in Andhra Pradesh, and the study area included villages and towns. The area is largely rural and semi-urban, with the majority of the population being involved in agriculture or agriculture related activities. The study area was divided in to 594 population units of discrete villages and towns, with a minimum population of 200. These were classified as rural low development, rural high development, or semi-urban, on the basis of infrastructure and development indicators including electricity, road and transport, and presence of a school and health facility. Infants were included in the study if they were BCG-vaccinated and were available for follow up for 2 years after enrollment, and a parent or guardian provided informed consent. Infants were randomized to active surveillance (regular home visits every 2 months for 2 years, which included anthropometry and a brief questionnaire to detect signs and symptoms of TB) or passive surveillance (cases were recorded if reported at health care facilities). The study did not involve any investigational product, but included a standardized protocol of TB diagnostic procedures if infants were suspected of having TB. Of a total of 7,424 recorded births in the study area, 4,878 fulfilled inclusion criteria and 4,382 (89.8%) were enrolled as participants. The study was approved by the institutional ethical review board of the St John's National Academy of Health Sciences and the Ministry of Health Screening Committee of the Government of India, in addition to which it underwent ethical review by a contracted Ethical Review Board of the Aeras Global TB Vaccine Foundation.

### Informed Consent Process

The field supervisors in the study team were responsible for obtaining informed consent. This team of 12 (with some replacements over the course of the study) was all male, each with a minimum of 12 years of school education. A minority of them had received a tertiary education. Study physicians and research nurses were not involved in the consent process, in case the power differential between health care providers and the study population influenced parents' decisions about whether to enrol their children in the study.

Study personnel were trained on ICH-GCP (International Conference on Harmonization - Good Clinical Practice) guidelines and on informed consent procedures as part of a professional development program. They were sensitized to the historical antecedents of current guidelines for the protection of human volunteers in clinical research. Specific to this research study, all personnel involved in obtaining consent were instructed to encourage questions from the parent providing consent and to note down the questions and comments at the back of the consent form if the questions went beyond simple clarifications of the contents of the informed consent form.

Parental consent was taken from any parent who was available at the home of potential participants. Initially a confidential setting was sought for the consent process. In practice, however, the discussions often took place in the presence of other family members or neighbours after the parent indicated his or her comfort with their presence. In this situation, the study team members were instructed to ensure that the decision to participate should be taken independently by the parent, without pressure from the other people present. Research coordinators and study managers would observe the informed consent process on supervisory visits and provide feedback to field supervisors to strengthen participatory consent practices.

The informed consent form was translated into colloquial Telugu, the local language. It was structured around questions, as the study team felt that this approach is more intuitive than a narrative consent form for both the study personnel and parents [8]. The main questions addressed in the consent form related to the identity of the sponsor; reasons for being selected for participation; study procedures including the TB diagnostic process; potential risks and benefits; confidentiality; research organizations' responsibilities; and, whom to contact for further information. After consent had been obtained, the socio-demographic characteristics of the parents were documented during a subsequent visit as part of the baseline information for each study subject.

### Analysis

Once recruitment for the research study was completed, the questions asked during the informed consent were coded into a range of different categories, loosely corresponding with the structure of the informed consent document. As many of the questions did not correspond exactly to the questions posed in the informed consent sheet, new codes were also created. The informed consent data were entered into the larger database for the study, which included socio-economic household characteristics.

The data were analysed in Statistical Package for the Social Sciences v. 17.0 (SPSS). Frequencies for type of question asked were calculated first. Subsequently, cross-tabulations with chi squared tests were conducted to identify statistically significant associations between asking a question during the consent discussion and a range of household social, economic and demographic variables. These included level of development of settlement in which the household was situated (low rural, high rural, semi-urban); mother's age; religion (Hindu, Muslim or other); Caste (Dalit/Harijan or other; Dalits/Harijans are the lowest Hindu caste in India and are typically socially and economically disadvantaged); and, mother's and father's education and occupation. Household construction material and household cooking fuel use were also assessed as indicators of socio economic status (SES); stone and brick construction was considered higher SES and all other constructions lower SES, and liquid petroleum gas was considered higher SES and all other types of cooking fuel use were considered lower SES. Other variables that were examined were household exposure to TB (we thought this might increase questions asked); both parents being present during consenting; and, which parent - mother or father - signed the consent form. Finally, the relationship between the fieldworker taking the consent and participation in the informed consent discussion was explored.

In the next stage of analysis, a multivariate logistic regression model was constructed to examine the effect of socio-economic and demographic variables on participation in the informed consent discussion. The binary outcome variable was "Did either parent ask a question during the informed consent discussion?" All the variables from the bivariate analysis that had shown significant association were included, except for the identity of the fieldworker. The reason for excluding the fieldworker variable was that our focus in the multivariate analysis was to identify participant related factors affecting participation in informed consent.

## Results

Out of the 4,382 children enrolled in the study, 4,095 (98.6%) of informed consent forms were signed by the child's mother. The child's father signed the consent form for 60 children (1.4%), and a guardian signed for 2 children. In only 287 cases (6%) were both parents present during the informed consent process. Out of these children, the mother signed the consent form for 271 (94.5%) children. In total, 590 out of 4,382 (13.4%) parents/guardians asked any question during the informed consent process.

Table 1 shows the range and distribution of questions asked during the informed consent process. Some households asked more than one question. Since each question was captured separately, the total number of questions (1,239) exceeds the number of households where questions were asked (590). The questions covered a range of issues, relating directly and indirectly to the content of the informed consent document. The highest proportion pertained to the nature of TB diagnostic tests to be performed on the child (23.7%), and possible risks to the child of being in the study (23.4%). The third most frequent question was about benefits to the child or family of participating in the study (17.8%), followed by general questions about TB infection, diagnosis and treatment (17.3%). Ninety-nine people (16.8%) enquired about the rationale or purpose of the study. Concerns that were expressed were about who to contact with any questions (16.4%), and how being in the study would affect treatment for other childhood illnesses (14.9%). Just over 10% of parents who asked any question asked whether free treatment would be available for the child and family members during the period that the research project was being conducted.

**Table 1:** Distribution of questions asked during informed consent discussions*

Nature of question	n	% of participants (n = 590)	% of questions (n = 1,239)
TB skin and sputum diagnostic tests	140	23.7	11.3
Risks to child of being in study	138	23.4	11.1
Benefits of being in study	105	17.8	8.5
Questions and comments about TB symptoms and treatment	102	17.3	8.2
What is the rationale for/purpose of the study	99	16.8	8.0
Duration of study	97	16.4	7.8
What are the study procedures	88	14.9	7.1
Confidentiality of child's information	62	10.5	5.0
Implications of participation for child's other health care needs	56	9.5	4.5
Questions and comments about other diseases	55	9.3	4.4
Who to contact with questions	53	9.0	4.3
Sponsors responsibility if child hurt during the study	52	8.8	4.2
Availability of treatment for child or family members	49	8.3	4.0
Who is the sponsor of the study	32	5.4	2.6
Eligibility for the study and who else participating	31	5.3	2.5
Costs of being in the study	25	4.2	2.0
Comments: Approval of the study or hospital	24	4.1	1.9
Any other question or comment	31	5.3	2.5

*Total*	*1239*	*100*	*100*

*Analysis of 1,239 questions asked from 590 out of 4,382 informed consent discussions in which a question was asked. Multiple questions were recorded in some consent discussions

Table 2 shows the bi-variate analysis of factors associated with asking any question(s) during the informed consent process. There were highly significant associations (p < 0.001) between asking questions and subjects' residence (semi-urban, rural high development or rural low development); higher parental education; both parents being present during the consent discussions; and the use of liquid petroleum gas as the main household cooking fuel (an indicator of higher socio-economic status where the majority of households 92.6% used solid cooking fuels).

**Table 2:** Characteristics of the study sample, and bi-variate analysis of participation in consent discussion

	Number (N = 4382)	No (%) within each category who asked question	**p-value**^**1**^
**Participation in Informed consent**			

Asked a question	590	590 (100)	
Did not ask a question	3792	0 (0)	

**Residence**			

Rural low development	1863	193 (10.4)	
Rural high development	2069	307 (14.8)	< 0.001
Semi-urban	450	90 (20)	

**Age of Mother (years)**			

17-19	326	58 (17.8)	
20-24	2710	355 (13.1)	0.119
25-29	1118	145 (13.0)	
30+	228	32 (14.0)	

**Religion**			

Hindu	3751	488 (13.0)	0.045
Muslim	614	101 (16.4)	
Other	17	1 (5.9)	

**Caste**			

Dalit/Harijan (Low caste)	850	99 (11.6)	0.046
Other	3532	491 (13.9)	

**Mother's education**			

Illiterate	1337	130 (9.7)	
Primary	1251	179 (14.3)	
Secondary	803	118 (14.7)	< 0.001
High School	729	116 (15.9)	
Higher Sec and College	262	47 (17.9)	

**Father's education**			

Illiterate	992	99 (10.0)	
Primary	1083	142 (13.1)	
Secondary	1021	143 (14.0)	0.001
High School	896	140 (15.6)	
Higher Sec and College	390	66 (16.9)	

**Mother's occupation**			

Professionals, managers, technicians	48	6 (12.5)	
Skilled workers (non-agriculture)	133	21 (15.8)	0.026
Skilled agricultural workers	1251	138 (11.0)	
Unskilled workers and unclassified	2950	425 (14.4)	

**Father's Occupation**			

Professionals, managers, technicians	132	23 (17.4)	
Skilled workers (non-agriculture)	860	143 (16.6)	0.008
Skilled agricultural workers	1578	200 (12.7)	
Unskilled workers and unclassified	1812	224 (12.4)	

**Construction of walls**			

Stone and Brick (higher SES)	3484	461 (13.2)	0.202
Other (lower SES)	898	129 (14.4)	

**Main Source of cooking fuel**			

Liquid Petroleum Gas (higher SES)	323	63 (19.5)	0.001
Other (lower SES)	4059	527 (13.0)	

**Household exposure to TB**			

No	4293	579 (13.5)	0.454
Yes	89	11 (12.4)	

**Number of parents at consenting**			

1	4095	451 (11.0)	< 0.001
2	287	139 (48.4)	

**Who signed consent form**			

Mother	4320	575 (13.3)	
Father	60	14 (23.3)	0.025
Other	2	1 (50.0)	

^1 ^Chi-squared test for significance of association with outcome variable (did participant's parent ask a question during the informed consent discussion?)

Other factors that were associated at the p < 0.05 level were religion (with Muslims being more likely to ask questions), caste (socially disadvantaged Dalits were less likely to ask questions), parental occupation, and which parent signed the consent form (more questions were asked when fathers signed). Factors that did not appear to have a statistically significant association with participation during the consent discussion were mother's age, the house construction material (another indicator of socio-economic status), and reported household exposure to TB.

Table 3 presents the results of the binary multiple logistic regression model to investigate correlates of participation in the informed consent process. The model included socio-demographic characteristics (residence, age of mother, religion and caste); household socio-economic characteristics (fuel used, construction of walls, father's occupation); parental characteristics (education level of parent who signed consent form); and, characteristics of the informed consent process (were one or two parents present at the discussion).

**Table 3:** Correlates of asking questions during informed consent process

Variable (*Comparison group for categorical variables*)	Odds Ratio (95% C.I.)	Confidence Interval
Residence (*low development rural area*)		
High development rural area*	1.376	1.126 - 1.681
Semi-urban area*	1.665	1.178 - 2.340
Mothers Age *(17-19yrs)*		
20-24 yrs	.779	.564 - 1.076
25-29 yrs	.777	.543 - 1.112
30+ yrs	.878	.527 - 1.463
Religion (Hindu)		
Muslim	1.106	.838 - 1.461
Other	.217	.027 - 1.773
Caste is Dalit or Harijan (low caste)	1.134	.878 - 1.463
Uses Liquid Petroleum Gas (higher SES)	1.066	.739 - 1.538
Walls of house made of brick or stone (higher SES)	1.148	.912 - 1.444
Father's occupation (non-skilled worker)		
Professional, manager or technician	1.285	.747 - 2.209
Skilled worker other than agriculture	1.087	.630 - 1.876
Skilled worker agriculture	1.164	.677 - 1.998
Education level of parent who signed consent *(illiterate)*		
Primary*	1.531	1.187 - 1.976
Secondary*	1.625	1.220 - 2.164
High school and above*	1.754	1.315 - 2.339
Both parents present at consenting*	7.319	5.693 - 9.547

* *p < 0.05*

Location had an effect on participation, with residents of low development rural areas least likely to ask questions, and residents of semi-urban areas most likely to ask questions. Education level was important, with those of a higher education level more likely to ask questions than those with a lower education level. The number of parents present during the consent discussion had a large effect on participation, with questions much more likely to be asked if both parents were present (Odds ratio = 7.5). All other variables were not significantly associated with asking questions; possibly because they are collinear with education and their effect is captured in the model by the education variable, or because of the small number of observations.

## Discussion

Institutional and government ethical review boards play an important role in ensuring that clinical and epidemiological research studies have well-defined and documented procedures for obtaining voluntary and informed consent from research participants. However, too often, little attention is paid to how the processes of obtaining consent are conducted. Important indicators of these processes are the extent to which research subjects participate in consent discussions and understand the nature of the research study. In developing countries, a further concern is that many of the principles of medical ethics have been developed within the social context of Europe and North America, and local considerations for obtaining consent may need to be better understood. The conduct of research studies in children poses further questions for how to establish procedures for obtaining parental consent that respect the autonomy of parents as well as protect the interests of the child. This study of participation in consent discussions in a community-based neonatal study contributes to the thinking about research engagement with participants, and consent for vulnerable populations.

Only a small fraction (13.5%) of parents asked any questions during the informed consent process. One possible reason for low participation could have been that a significant number of doubts and concerns were clarified during the prior community information sessions (n = 196). Community sessions had been held in schools and village meeting spaces, and were attended by students, teachers and parents. However, when we analysed the focus of the 176 questions that has been recorded over the course of the community information sessions, the majority of these (61.3%) were related to doubts about signs and symptoms of TB. The relatively few questions asked about the study procedures indicate that the community information sessions are not likely to be a major explanatory factor for low participation in the informed consent process. Another possible reason for low participation is that the study was being conducted under the auspices of an organization that has provided charitable health services in the community for more than 30 years; this may have led to an implicit trust in the beneficence of any study being conducted, associated with lower likelihood of expressing doubt or seeking clarification.

If participation is taken as an indicator of the extent to which consent is informed, it is a matter of some concern that so few questions were asked during the informed consent discussions, despite significant efforts being made on the part of the research team to encourage open discussion and questioning, and allow households to be involved in the consent process. This may well result in poor understanding of the nature of the research study, as found in other settings [10,18,22]. The analysis identified location, education level and both parents being present during the consent process as the most important factors affecting participation in the consent process. Those in more developed areas (rural areas with high development and semi-urban areas) were more likely to ask questions, perhaps indicating a greater exposure to health education, media and information about research. This could be associated with greater ability to formulate questions and confidence to ask them. The effect of education may be related both to those who are educated having a better understanding of research processes (and therefore having a better idea of which questions to ask), and having greater confidence to ask questions. A study of the quality of informed consent in South Africa reported similar findings with regard to education [13].

The fact that more questions were asked in our study when both parents were present may indicate a greater confidence to ask questions in a group setting. However, some of this effect may also be gender-related, with fathers more likely to ask questions to researchers than mothers (in the vast majority of cases where only one parent was present, this was the mother). This effect may be even more pronounced in our study, since all of the study team members obtaining consent were male. These findings suggest that further research is needed to understand the extent to which participation in informed consent discussions is influenced by the gender of both the research participant and study team members.

There are some limitations to this study. First, asking questions during the consent process is taken as an indicator of the extent to which consent is informed; this may not necessarily correspond with actual knowledge and understanding of the research process. Second, we made the assumption that field supervisors were equally likely to write down any questions asked during the consent process. It is possible, however, that the likelihood of recording questions asked was related to the ability/interest of fieldworkers. We believe that the periodic supervision by the project coordinator of field supervisors for the consent process and recording of questions strengthens the validity of data. Another issue is the possible relationship between fieldworker characteristics and parents' comfort asking questions. In bi-variate analysis, we found an association between the fieldworker who took the consent and whether any question was asked; however, we did not have sufficient information on fieldworker characteristics (such as communication style, educational background, previous experience with research) to conduct further analysis of this important area. Given that previous research in South Africa found that the quality of informed consent was better when consenters had more experience [13], and other authors have suggested that research workers do not always have the necessary communications skills to encourage open discussion and questioning of a research project. [7], this is an important area for future research. A final limitation is that the findings are limited to one study. However, the number of cases is large (n = 4382), and the data can be said to be broadly representative of rural India.

## Conclusions

Our findings provide two important lessons for planning the process of informed consent for enrolling children in research studies in developing country settings. First, given that socio-economic status and education are significant predictors of whether any questions were asked during the informed consent process, it is critical to put more effort into considering innovative approaches for making discussions during the consent process more interactive. This may involve including pictures, stories, and some test of understanding of the study aims and procedures. A primary concern for researchers should be to make the process more accessible and meaningful for research participants of all socio-economic groups, and to raise the level of research literacy within the community. Apart from carefully considering the content of informed consent procedures, the impact of training research team members in communication skills (for example, limited jargon and pausing after every sentence to allow opportunities for questions) on research subjects' participation and comprehension should be examined in future research. The collection of data on participation can also serve as a useful monitoring indicator for quality of informed consent processes within research studies.

A second issue for consideration in conducting research with children is the amount of effort that should be put into seeking two parents' participation in the consent process, where possible. Although mothers are the primary caregivers of children and are likely to be easier to reach in a household study, our analysis showed that fathers were much more likely to ask questions during the informed consent process. In order to promote consent that is truly informed, it may be desirable to create more opportunities for fathers to discuss research studies with research workers. Given that fathers are harder to reach during working hours, this could increase the costs of the consent process significantly, which can be a deterrent where research funds are constrained. If mothers are targeted as the primary consenters for children's studies, participation in consent may be increased by having female research workers conduct the consent discussion; it would be valuable to conduct further research to test this hypothesis.

Finally, this research points to the importance of generating more empirical evidence on consent and understanding of informed consent in different settings, and of building quality assurance of these procedures into research protocols. With the growing volume of health research being conducted across different social, cultural and economic settings, it is critical for researchers to continue to introspect on ethical aspects of their work and increase their engagement with research participants.

## Competing interests

The authors declare that they have no competing interests.

## Authors' contributions

DR conducted the primary analysis and drafted the manuscript. NJ was involved in conceptualizing the study, coordinated the data collection, contributed to the interpretation of the data, and reviewed the manuscript. LG was involved in conceptualizing the study and reviewing the manuscript. SB was involved in interpreting the data and reviewing the manuscript. HG was involved in conceptualizing the study, interpreting the data, and reviewing the manuscript. MV was involved in conceptualizing the study, contributed to the analysis and interpretation of the data, and reviewed the manuscript. All authors read and approved the manuscript

## Pre-publication history

The pre-publication history for this paper can be accessed here:

http://www.biomedcentral.com/1472-6939/12/3/prepub
